# Barriers and facilitators of kangaroo mother care adoption in five Chinese hospitals: a qualitative study

**DOI:** 10.1186/s12889-020-09337-6

**Published:** 2020-08-13

**Authors:** Jieya Yue, Jun Liu, Sarah Williams, Bo Zhang, Yingxi Zhao, Qiannan Zhang, Lin Zhang, Xin Liu, Stephen Wall, Greta Wetzel, Gengli Zhao, Jennifer Bouey

**Affiliations:** 1 Peking University First Hospital, 1 Xi’anmen St, Xicheng, Beijing, China; 2 Save the Children UK, London, UK; 3 Nuffield Department of Medicine, University of Oxford, Oxford, UK; 4 Save the Children, Chengdu, China; 5 Save the Children Saving Newborn Lives, Washington, DC USA; 6 Department of International Health, School of Nursing and Health studies, Georgetown University, Washington, DC USA

**Keywords:** Kangaroo mother care, Qualitative, Implementation research, China

## Abstract

**Background:**

Kangaroo mother care (KMC) has been proved to be a safe and cost-effective standard of care for preterm babies. China hasn’t adopted the KMC practice widely until recently. We aim to assess barriers and facilitators of KMC adoption in neonatal intensive care units (NICUs) and postnatal wards in China.

**Methods:**

We conducted clinical observations and semi-structured interviews with nurses, physicians, and parents who performed KMC in seven NICUs and postnatal wards housed in five hospitals in different provinces of China between August and September 2018. The interviews provided first-hand stakeholder perspectives on barriers and facilitators of KMC implementation and sustainability. We further explored health system’s readiness and families’ willingness to sustain KMC practice following its pilot introduction. We coded data for emerging themes related to financial barriers, parent- and hospital-level perceived barriers, and facilitators of KMC adoption, specifically those unique in the Chinese context.

**Results:**

Five hospitals with KMC pilot programs were selected for clinical observations and 38 semi-structured interviews were conducted. Common cultural barriers included concerns with the conflict with traditional postpartum confinement (Zuo-yue-zi) practice and grandparents’ resistance, while a strong family support is a facilitator for KMC adoption. Some parents reported anxiety and guilt associated with having a preterm baby, which can be a parental-level barrier to KMC. Hospital-level factors such as fear of nosocomial infection and shortage of staff and spaces impeded the KMC implementation, and supportive community and peer group organized by the hospital contributed to KMC uptake. Financial barriers included lodging costs for caregivers and supply costs for hospitals.

**Conclusions:**

We provided a comprehensive in-depth report on the multi-level KMC barriers and facilitators in China. We recommend policy interventions specifically addressing these barriers and facilitators and increase family and peer support to improve KMC adoption in China. We also recommend that well-designed local cultural and economic feasibility and acceptability studies should be conducted before the KMC uptake.

## Background

Kangaroo mother care (KMC) is recommended by the World Health Organization (WHO) as a standard for the care of preterm newborns [1]. Key features of KMC include early, continuous, and extended skin-to-skin contact between infant and caregiver; exclusive breastfeeding. KMC practice is often associated with early discharge from hospital and necessary support for caregiver and infant at home [2]. WHO recommend that the continuation of KMC until the newborn’s gestational age reaches term (gestational age around 40 weeks) or the weight reaches 2500 g [2].

KMC was initially proposed as an alternative to the conventional contemporary method of care for low-birthweight infants and was introduced to some low-income countries including Bangladesh, Malawi, Nepal, etc. in the 1980s and 1990s [3]. In these countries, the prevalence of prematurity/low birth weight is high while medical resources are limited, KMC was performed as an alternative to incubators or warmers [4]. Systematic reviews of KMC studies reported that the practice of continuous or intermittent KMC was associated with a significant reduction in the risk of neonatal mortality as well as sepsis, hypoglycaemia, and hypothermia. KMC also has multiple benefits for newborns and their families, including improved duration of breastfeeding, weight gain and physiological response [5], supporting maternal-infant bonding and child development, improving longer term neuro-behavioural, psychomotor development and brain maturation [6–8]. Currently, KMC is considered one of the most cost-effective interventions to promote the well-being of preterm infants, and has been widely adopted in many countries, such as in Africa, Asia, America, Europe, and Latin American and the Caribbean [6–8]. It was estimated that KMC was practiced in some form (mostly intermittent) in 82% of all neonatal intensive care units (NICUs) in the United States [9].

China is a populous country with nearly 1.4 billion people and more than 15 million annual births, of which 99.9% are facility-based deliveries in one of China’s 10,000 primary hospitals, 9000 secondary hospitals and 2500 tertiary hospitals (including around 800 maternal and child health specialized hospitals) [10]. China has successfully reduced maternal and neonatal mortality through different health and social sector initiatives and investments including improvement of legislation and policy, surveillance and interventions such as the Baby Friendly Hospital Initiative [11]. Additionally, the basic public health services at the primary care level include disease screening and management for all newborns and follow-up for high-risk newborns [12], while meanwhile allowing referral of high-risk newborns to secondary and tertiary hospital networks as needed. Currently, neonatal deaths account for over 50% of deaths among children under 5 years of age in China, among which complications of prematurity is the leading cause of death [13]. According to the 2019 China Health Statistics Yearbook, the neonatal mortality rate in 2018 was about 3.9 per 1000 live births and 3.13% of newborns weight less than 2500 g at birth [10]. A recent nationwide survey across 25 provinces in China estimated the incidence of preterm birth was 7.3% [14]. According to a systematic estimation in 2010, China contributes about 7.8% of global preterm births (1.7 million), second only to India (3.5 billion) [15].

Despite the high number of premature babies in China and the many benefits of KMC for premature infants, especially those with low-birthweight, KMC has not been widely used or promoted in China. The previous technical guidelines for preterm infants issued by the Chinese Medical Association Paediatrics Society in 2005 listed recommendations for management at birth, nutritional management, nursing and follow-up care, but did not include KMC [16]. One of the main reasons is that in most NICUs in China, parents are denied entry or visits are limited to a few minutes per week [17]. China’s construction and management of neonatal wards guideline issued in 2009 stipulates that access to neonatal wards for non-staff members should be strictly limited [18]. Therefore, most NICUs allow very limited parental access, making KMC difficult to implement, this separation of mother and baby hinders bonding and increases parents’ anxiety. Stable late preterm infants generally room-in on the postnatal ward with their mothers, as they stay only one to four days, the opportunity for parents to gain confidence in the practice of KMC prior to discharge is limited.

Since 2014, China’s Premature Birth Intervention Programme has worked to raise awareness and promote the implementation of KMC amongst its network of 50 hospitals across China. Technical support included international expert meetings and KMC stakeholder workshops involving nurses, paediatricians and other health workers at their respective hospitals. Under this programme, a network of self-selected hospitals from different provinces in China participated in a pilot to implement standardized KMC guidelines in their NICUs and postnatal wards in order to assess the feasibility and factors influencing adoption of intermittent KMC in the Chinese context. Due to space and human resource constraints, intermittent rather than continuous KMC was practiced in the hospitals. It was recommended to parents that they continue to provide intermittent KMC to their newborns post discharge. KMC was largely accepted in China’s hospitals more rapidly than was initially anticipated, and the latest “Preterm Infants Health Care Service Guideline” published by the National Health Commission in 2017 [19] and the “Action Plan for Healthy Children (2018-2020)” [20] stipulated that kangaroo care should be promoted to improve the quality of life for premature babies. We undertook this study to inform the further sustain and scale up of KMC across China through a better understanding of the barriers and facilitators to KMC in the Chinese context.

Previous systematic reviews suggested barriers for KMC practice in developing and developed countries included “issues with the facility environment /resources,” “negative impressions of staff attitudes or interactions with staff,” “lack of help with KMC practice or other obligations,” “low awareness of KMC/ infant health.” “maternal pain/fatigue,” “actual increased staff workload” and “concerns about other medical conditions”. The main facilitators to the practice of KMC included “support from hospital management”, “mother-infant attachment” and “support from family, friends and other mentors” [21–24]. Despite there are studies published on barriers and facilitators of implementing KMC in many Asian countries including Nepal, India, Pakistan, Bangladesh, Vietnam, Philippines and Indonesia [3, 22, 25, 26], we believe that China may have some unique barriers and facilitators to the practice of KMC due to its specific policies, resources and traditional culture and we are not aware of any prior published studies in China examining these barriers and facilitators.

Using data from clinical observations and semi-structured interviews conducted in five hospitals, we aimed to 1) establish a conceptual framework to analyse the barriers and facilitators to KMC scale-up; and 2) provide recommendations for KMC adoption in Chinese hospitals to ensure sustainability and scale up.

## Methods

The present study is part of a larger project aimed to investigate the implementation of KMC in the neonatal and postnatal wards of piloting hospitals in China, after the development of standardized guidelines for KMC. In the current study, we collected data from clinical observation and semi-structured interviews in seven NICUs and postnatal wards housed in five hospitals and analysed the barriers and facilitators of KMC implementation in these hospitals.

### Study setting

The research was conducted in seven NICUs and postnatal wards across five tertiary hospitals from different provinces (Fujian, Guangdong, Hunan, Shaanxi, Shandong) in China between August and September 2018. These hospitals are self-selected for this research and are located in five provinces which vary in geographic locations, economic development, and cultural custom. Among the five hospitals, hospital B is a general hospital, and the other four hospitals are specialized maternal and child hospitals. All hospitals had not formally adopted KMC prior to the implementation study. During the implementation phase, hospital A and D piloted KMC both in NICUs and postnatal wards, while the other three hospitals implemented KMC only in NICU wards. Prior to KMC implementation, all of the NICUs in the pilot hospitals only allowed very limited parental access visitation, separating newborns and mothers for extended periods; whereas the postnatal wards allowed rooming-in, where newborns and mothers stay together.

### Participants

The KMC study coordinators, usually head nurses of the wards, helped identify medical staff and parents that were involved in KMC implementation or had KMC experience. In each study site, we recruited a mix of paediatricians/obstetricians, nurses, and parents from the NICUs and postnatal wards. We included full-time nurses and paediatricians/obstetricians on site, and parents who had experience with intermittent KMC. Finally, there were 38 participants recruited from seven hospital units including five NICUs and two postnatal wards.

### Data collection

Data collection was conducted by six research assistants, mainly hospital staff and project officers with prior KMC experience. Prior to data collection, all team members received systematic training on qualitative research, especially on data collection processes, including observation and interview techniques. All study team members were involved in data collection, transcription, data cleaning and analysis. All interviewers were female.

Data collection processes included clinical observations and semi-structured interviews with study participants. Use of observation in implementation research is common as a means to collect detailed information on processes and work roles, as well as insights into organizational values, norms and communication. The study team conducted observations to understand the general clinical activities at the beginning of the site visit to each hospital, including basic information about the wards (e.g. the number of rooms/beds/patients/staff) and the implementation of KMC (e.g. supplies for KMC, work patterns, medical staff roles, clinical process), and completed a standard observation form (see additional file 1). The study team designed the observation form and made minor revisions during the project according to the experience of observation.

The study team conducted and recorded, semi-structured interviews with physician and nursing staff and parents. The semi-structured interview tool was informed by the Consolidated Framework for Implementation Research (CFIR), commonly used in implementation-focused needs assessment, which provides a structured guide of five domains (i.e., intervention characteristics, outer setting, inner setting, characteristics of individuals, and process), each with accompanying constructs associated with the process of effective intervention implementation [27, 28]. The study team designed the initial interview guide according to the CFIR framework, and after pilot interviews and internal discussions, the interview outline was revised and adjusted, combining broad, open-ended questions with targeted, prompting questions, mainly to ensure the questions could be easily understood by the interviewees (see additional file 2). Interviews with a variety of stakeholders, including physicians, nurses and mothers, allowed the study team to gain multiple perspectives and validate findings. Interviews with clinical staff gathered their perspectives on barriers and facilitators of KMC program implementation and sustainability and helped to assess the readiness and willingness to continue KMC program post-intervention. Interviews with parents who provided KMC focused on their infants’ clinical condition, their knowledge of KMC, confidence of practicing KMC, and perceived benefits and barriers for KMC practice. Interviews were conducted over one to two days at each site. Each interview lasted about 30–40 min, and all interviews were conducted in Mandarin. The study team members conducted interviews in private rooms at the hospital. All the tools were piloted in the first hospital unit for validity and modified before the full data collection.

### Data analysis

Information from clinical observations were compiled for comparison and analysis. Semi-structured interviews conducted in Mandarin were audiotaped and transcribed by translational software verbatim for the initial transcripts. These initial transcripts were manually verified against the recording for fidelity in transcription by the interviewers. The verified transcripts were then imported as PDF files into a qualitative data analysis software Dedoose to facilitate data coding, text searching, and analysis (Dedoose Version 8.1.8, 2018). Descriptor data on the interviewee’s age, gender, interview site, position at the hospital (or parent information), tenure at the hospital was added to the Dedoose project and linked to each transcript for further analysis. Data was systematically examined for emerging codes, themes, and patterns. To ensure inter-coder agreement, two study team members developed open coding schemes independently on a subset of interviews, then made comparisons to derive consensus on the coding strategy. The coders independently coded another subset of interviews to ensure consistency of the implementation of the coding strategy. In this paper, we focused on the barriers and facilitators of KMC adoption, specifically barriers and facilitators unique in the Chinese context and less commonly reported in previous systematic reviews. Barriers to KMC adoption refers to the factors that can potentially hinder KMC implementation roll-out or make sustainability of KMC practice more difficult. Facilitators are the positive factors that either assist initial KMC implementation or enhance the KMC sustainability in China. A conceptual framework focusing on different level barriers and facilitating factors was also presented in the result section. Relevant themes related to knowledge of KMC, perceptions of KMC benefits, KMC implementation barriers, KMC’s impact on workflow and resources, inner and outer environmental factors that influenced the dynamics of KMC adoption emerged as the most relevant themes under each level. Through axial coding and developing the conceptual framework, we extracted direct quotes of the hospital staff and parents into excerpts, organized excerpts into categories and themes. Preliminary findings were also presented to all other authors who were not directly involved in data collection at an internal workshop to triangulate and increase trustworthiness of the findings.

### Ethical approval

Ethical approval was obtained from Peking University First Hospital Biomedical Research Ethics Committee. Verbal informed consent was obtained from all participants.

## Results

### General characteristics of the study units and participants

We analysed the general characteristics of the 38 participants, and the distribution of respondents in various hospitals is shown in Table 1. Detailed characteristics of respondents are shown in eTable 1 and eTable 2 in additional file 3 and the results of clinical observation including the general characteristic of the hospital units are shown in additional file 4. As hospitals A and D had both NICUs and postnatal wards participating in KMC, three nurses from each ward were interviewed. All 18 nurses and six of the ten physicians interviewed were women, while only one father was sampled among the ten parents of pre-term babies although he was not the only father providing KMC. Of the nurses and the physicians interviewed, half were from the NICU wards and half from the postnatal wards. 30% of the parents were first time parents. For physicians included in the study, 60% were junior and 40% were senior in clinical ranking; as for nurses, almost half were junior and half were senior in ranking.

**Table 1:** Distribution of respondents in various hospitals

Study site	Unit included	Interviewee
Hospital A	NICU + PNW	6 nurses, 2 doctors, 2 parents
Hospital B	NICU	2 nurses, 2 doctors, 2 parents
Hospital C	NICU	2 nurses, 2 doctors, 2 parents
Hospital D	NICU + PNW	6 nurses, 2 doctors, 2 parents
Hospital E	NICU	2 nurses, 2 doctors, 2 parents

Note: NICU, neonatal intensive care unit; PNW, postnatal wards

The observation in five hospitals indicated that the nurse/bed ratio ranged from 1:2.7 to 1:3.8. In one hospital, parents could perform KMC at any time in NICU wards and postnatal wards, while in other four hospitals, parents pre-booked morning or afternoon KMC time slots. Both of the two postnatal wards were room-in, while the NICU wards allowed limited parents access and provided video visitation or corridor visitation for parents who did not perform KMC. KMC chairs were provided for parents, and most of the parents wore their own clothes when performing KMC. Medical staff provided KMC introduction, posture adjustment, feeding instruction, temperature taking for parents during KMC sessions.

### Summary of the barriers and enablers

In this study, we categorized the facilitators and barriers of adopting KMC into different levels, including cultural level, hospital level, parental level, and financial level. A preliminary conceptual framework focusing on different level barriers and facilitators is presented in Table 2. In the results section, we specifically describe those barriers and facilitators unique in the Chinese context and less commonly reported in previous systematic reviews.

**Table 2:** Conceptual framework of barriers and facilitators for kangaroo mother care adoption in China

Cultural barriers	Hospital barriers	Parental barriers	Financial barriers
• Postpartum confinement • Grandparent resistance • Fear of sweating in summer/ cold in winter	• Fear of nosocomial infection • Shortage of staff • Lack of space • Parents monitoring work • Increased workload	• Anxiety/ fear of being in hospital • Loss of privacy/ lack of private space • Perfectionist psyche • Maternal guilt associated with preterm birth	• Lodging costs • Loss of work time • Transportation/ traffic • KMC supply costs to • hospitals
**Cultural facilitators**	**Hospital facilitators**	**Parental facilitators**	**Financial facilitators**
• Strong family support	• Hospital readiness: chairs, training, nurses • Leadership of the hospital’s administration/ motivation system • Creating supportive community: social media group	• Benefits to mothers • Benefits to newborns	• Parents with good financial status

#### Postpartum confinement

Most families in China still follow the deeply rooted “Zuo-Yue-Zi” (postpartum confinement) culture, in which the new mother has to stay indoors (mostly in bed) without doing any housework, consume only warm water and food, and not take a shower in order to reduce the possibility of long-term complications related to delivery. While this cultural practice could potentially bring health benefits to mothers and newborns, it was mentioned as a major cause of hesitation of mothers to participate in KMC during the first month after delivery, especially as they fear being criticized by their mothers, in-laws, and other caregivers if they wanted to forgo the postpartum confinement practice. These mothers often ended up delaying KMC initiation or asking the father or even their parents to act as a substitute for KMC. The relatives can provide KMC but certainly cannot breastfeed the baby at the same time.

*The biggest challenge, as mentioned earlier, is that they may be unwilling to come during postpartum confinement. Some would postpone rather than saying that they will not come. Some say I might come perhaps after my confinement period or the first ten or twenty days after childbirth. (Hospital E, NICU nurse 34).*

*As many Chinese people are very traditional, they are reluctant to come outdoor worrying that it’s bad for their recovery. (Hospital C, NICU nurse 18).*

#### Grandparents’ resistance to KMC

In addition, many grandparents were not used to the idea of allowing newborn babies to remain naked with the mother; they were concerned that sweating in summer and cold temperatures in winter might harm the babies during KMC practice. Some mothers new to KMC were also nervous about touching their babies’ delicate skin.

*… My concern is that there might be (resistance) during the winter season. Our program started in summer, thus the parents accepted it well. If we promoted KMC in winter, perhaps some grandma and elders would be worried that the babies catch cold. This depends on their acceptance of this practice. Young parents can accept it easily. (Hospital A, Obstetric nurse 7).*

#### Anxiety of carrying for preterm baby

Parents reported anxiety of preterm babies’ “fragility” and were afraid to touch their newborns, especially if their newborn had unstable vital signs. For example, in NICUs, most parents have to hold the baby facing the medical monitors, some babies might have non-invasive ventilators. As the babies’ vital signs change during KMC, parents become more hesitant to perform KMC.

*It was difficult for me at first, because the baby was so small, I dared not to touch it … as I felt it’s inappropriate no matter where I touch. (Hospital D, parent 32).*

*(Some parents) were worried that the oxyhaemoglobin saturation of some babies dropped to too low and dared not to hug it. Later perhaps the condition became stable and we explained it to them again and again, so they hugged it again and contacted it. However, once they hugged it and the oxyhaemoglobin saturation dropped again, they were reluctant to do this again. (Hospital B, NICU doctor 13)*

#### Lack of private space

Another side-effect of having people other than the mother performing KMC was that there’s a lack of private spaces for mothers and fathers. As NICUs are crowded with six to ten people including both men and women, mothers reported that they felt embarrassed when exposing their body during KMC, especially in KMC rooms where curtains or dividers between the chairs are unavailable. Hospitals that provided curtains between KMC chairs/beds received much better remarks on this aspect. Some mothers expressed the embarrassment to be naked in front of medical staff especially the male physicians. Embarrassment was also a factor for fathers as when providing KMC they had to be naked in front of women.

*In the postnatal wards, even though we have a curtain, it’s difficult to protect the privacy if when the family members come and go. So, I think their privacy cannot be protected, this is a limitation for sure. (Hospital D, Obstetric nurse 30).*

*There are too many people in different wards, and some are male, the baby is naked, and I am almost naked in the upper part, it’s inconvenient. (Hospital A, parent 31).*

#### Maternal guilt associated with preterm birth

Some mothers also considered that delivering a pre-term baby was a “failure” due to belief in perfectionism. These mothers of low-birth-weight preterm babies often felt stigmatized, stressed, and depressed, which often impeded their willingness to face and hold the baby every day.

*The mother had anxiety, she always asked her mother to perform KMC and refused to face the fact … She had huge societal pressure and refused to face a 30 gestational week or 1000 g, 900 g baby, she cannot face it … At first, she was reluctant to enter NICU, as she could not believe that she had given birth to such a tiny baby. (Hospital A, NICU doctor 4).*

#### Fear of nosocomial infection

While physicians are less involved in the daily implementation of KMC, their discussions on the barriers for KMC focused on traditional concerns about nosocomial infection in the NICUs. Some nurses and physicians said they were only convinced of KMC’s benefit after the hospital routine statistics showed the infections in the hospital did not increase after KMC implementation. Some physicians also expressed concerns about the pressure on hospital resources when parents practicing KMC used hospital-provided diapers, water stations, and bathrooms.

*When KMC practice started, hospital administration was prioritized over medical practice, thus patients were not allowed to enter the wards as it may increase the risk of cross infection and waste the measures for disinfection and isolation. There was high resistance due to infection control. (Hospital A, NICU doctor 28).*

Some nurses also expressed concerns about the increased chance of bacterial infection in the crowded environment, especially when mothers do not shower due to “Zuo-Yue-Zi” (postpartum confinement).

*Some mothers do not wash their head or shower during postpartum confinement. We told them to wash and dressed neatly and cleanly. Still, some parents have not cleaned themselves well when they come. (Hospital C, NICU nurse 17).*

#### Parents monitoring work

Due to the limited visitation policies of NICUs in China, nurses and physicians are not used to performing clinical procedures on newborns whilst their parents are observing. Medical staff are concerned that parents may monitor their routine procedures, including injections, and their emergency treatment and are worried that conflict may arise between parents and themselves, if parents consider medical procedures to be too “brutal”. This is a reflection of the tense doctor-patient relationship in China. Nonetheless, medical staff and parents reported that after parents became familiar with the routine work of medical staff, they become less anxious and many reported better relationships with doctors and nurses.

*Our wards were “closed-door” operating before and the nurses were afraid when family members came in (for KMC), especially the new nurses – they were very nervous, they did not know how to talk to them or how communicate the disease condition to them, because the condition that the nurses could explain to them is very limited, just some very normal things, so family members may had less trust in the nurses. (Hospital A, NICU nurse 9).*

#### Leadership of the hospital’s administration/motivation system

Many nurses and doctors attributed their enthusiasm for KMC to the leadership of senior hospital administrators. In these circumstances, administrators supported KMC and ensured provision of training and discussion at whole-hospital and individual-department levels, they provided necessary equipment, including reclining chairs, KMC rooms, and identified an existing nurse to specialize in KMC. Routine supervision on KMC implementation was reported as one of the incentives for the medical staff to continue KMC work.

*Parents’ understanding, support of from hospital leadership, the cooperation of doctors and parents had assisted us greatly in our work. We have only one or two from the beginning. Three or four beds has been expanded to six beds, and there will be more opportunities to expand in the future. (Hospital C, NICU nurse 18).*

#### Creating supportive community: social media group

WeChat is the most popular form of social media in China, many hospitals use it for health promotion and education as well as connecting parents of preterm infants through establishing WeChat groups (a Chinese social media platform allowing group conversation). The former provides a platform where parents can understand the benefits of KMC, and the latter enables parents to communicate with each other and share KMC experiences.

*In terms of promotion, I also think of WeChat accounts … you bring together the parents of premature babies … Through these platforms you promote the theory (of KMC), which will facilitate the actual operation later. If parents do not have any knowledge of KMC and are directly taught to perform KMC, it would be hard. They can learn from the pictures or videos and know what’s their role in KMC. When they perform, they could be less anxious. (Hospital A, NICU nurse 9).*

#### Parents with good financial status

Some of the parents lived far away from the hospital and reported long commute times. Some parents even had to rent a house near the hospital. Therefore, for families with poor economic conditions, the extra cost of transportation and rent for KMC may become a burden. Some families with good financial status considered the benefit of KMC outweighed the cost.

*So some parents considered the money, the rent, so they may live at a place farther. Moreover, the accommodation nearby is expensive. Some families have already spent a lot on assisted reproduction, so they would consider saving money. (Hospital B, NICU nurse 11).*

*The parents are willing to rent a house in the village near the hospital, because compared with the spending for treatment of some infection, the housing rent is nothing. (Hospital A, NICU doctor 4).*

## Discussion

This is the first study to assess the barriers and facilitators to KMC in China. Our analysis uncovered barriers and facilitators at cultural, hospital, parental and financial levels. Some of these barriers and facilitators appear unique to the Chinese setting. If KMC is to be successfully scaled up in China it is necessary that these factors be taken into account when planning KMC promotion and implementation strategies.

### Barriers and facilitators at cultural level

Previous studies identified cultural factors as important influencers of KMC practice, for example some cultures considered skin-to-skin contact between infants and their carers to be inappropriate [24]. Specific cultural barriers found in our study include the practice of postpartum confinement and resistance to KMC from grandparents and community members rooted in tradition. Postpartum confinement is a traditional custom in China [29, 30], and it is also seen in other countries including India and Bangladesh [31, 32]. In China and India, most mothers are advised to stay at home after delivery [30, 32]. While this practice has potential health benefits for mothers and newborns, it also led to mothers and family’s being hesitant to initiate KMC. This especially applied when newborns were being cared for on NICUs and their mothers had been discharged home. Once home the practice of postpartum confinement prohibited mothers from traveling to the hospital to provide KMC.

A review of the literature on barriers and facilitators to KMC showed that studies in other countries identified country or context specific cultural barriers, for example, the practice of carrying babies on the back instead of the front is commonly reported as a barrier in many countries [33, 34], and in Ghana and Bangladesh early bathing of newborns was reported as a barrier to KMC and skin to skin contact after birth [33, 34]. Understanding these context specific barriers allows the promotion of KMC to be tailored to the needs of the population. For example our study found that resistance from grandparents to KMC was a barrier to practice, KMC promotion could target grandparents. A study in Malawi looked at the effect of educating grandparents about maternal and newborn health, grandparents subsequently provided individual and group counselling in their villages using songs, drama and poems, which was successful in bringing about behaviour change in maternal and neonatal care [33, 34]. As with our study, previous studies have shown that mothers need support and assistance from family members during KMC provision, especially with household chores [31, 34, 35]. If mothers receive adequate family support they appear more willing to perform KMC. When introducing KMC to parents and family members, additional health promotion and activities targeted at grandparents and other family members informing them of the benefits of KMC and the support required by mothers might lead to an increase in KMC uptake.

### Barriers and facilitators at hospital level

Studies from other countries have found that at hospital and health system level, the absence of leadership, inflexible visitation policies for parents, insufficient staffing levels, and insufficient training were barriers to KMC [3]. Evidence from other Asian countries shows that leadership and governance, the presence of essential medical products, the health workforce and health financing were important factors influencing the adoption of KMC [22, 25, 36]. For example a study of the introduction of KMC in Indonesian hospitals found that support from central government, hospital management, staff acceptance and training were identified as key facilitators for KMC implementation, these factors were also raised by clinical staff in our study [25, 36].

Our study similar to previously reported studies found lack of space for KMC provision to be a primary concern [34, 37, 38]. Population density and large numbers of deliveries especially at tertiary-level mean that hospitals are constantly struggling to secure sufficient space for NICUs. Accommodating rooms for KMC, chairs and maintaining sufficient privacy for the parents providing KMC in NICUs add an additional burden. The crowding and noise created by inviting parents and family members to NICUs discouraged nurses from introducing KMC. Due to space and human resource constraints, intermittent rather than continuous KMC was practiced in hospitals in China. For parents and other care givers lack of space and privacy in facilities presented obstacles to KMC adoption, a commonly observed barrier in studies from other countries [24, 39]. Lack of space meant that parents had to wait for NICU staff to schedule time for their provision of KMC and when parents were permitted on to NICUs to provide KMC the length of time they could stay was often limited. Mothers reported feeling uncomfortable and exposed as medical staff continued to come and go during KMC provision, there was no private space for mothers to provide KMC and hospitals lacked the space that would have enabled mothers to remain in the hospital with their newborns providing KMC.

Additionally, the acute shortage of paediatricians and paediatric nurses across China is a major concern [40, 41]. The nurse to preterm baby ratio in China is relatively low when compared to other high-income countries such as the United States [42, 43]. The already overwhelming workload of nurses can be further taxed by the necessity to train and supervise mothers providing KMC, additionally due to a lack of relevant systems, most hospitals relied on nurses to record research, evaluation and monitoring data for KMC. The challenge of human resources, fear of increased workload, record keeping and data collection are factors that have also been reported as barriers to KMC in countries, such as Malawi and Indonesia [23, 25]. However, it has also been shown that after an initial increase in workload at the start of KMC implementation KMC reduces nurses workload [25].

It’s noteworthy that lack of resources was not mentioned as a barrier for KMC adoption in China. A multi-country analysis of health system bottlenecks from 12 African and Asian countries reported poor availability of basic supplies for KMC in health facilities as barriers [22]. That this was not mentioned in our study may be due to the projects development of a standard list and budget for equipment needed to support KMC and the health systems ability to purchase this equipment. Additionally based on perceived need some hospitals even designed and procured clothes and other supplies to support the provision of KMC.

While many of the above-mentioned barriers are seen in other low- to middle-income countries [21, 34], what is unique to China is the restrictive parental visiting policies of the majority of NICUs where parents are denied entry and therefore access to their newborn [17]. The concept of closed-off management originated from hospitals’ concern about nosocomial infection. As many mothers do not shower due to postpartum confinement, and the NICUs are often overcrowded, hospital administrators, physicians and nurses initially expressed huge concern over KMC and feared it may lead to the spread of nosocomial infection. After advocacy, visits to hospitals overseas and individual experience of managing the first few cases of KMC practice, clinical staff were gradually convinced that KMC’s benefits outweigh the fear of nosocomial infection and accepted and promoted KMC.

Our analysis suggested that hospital-led peer support groups and educational platforms enabled KMC adoption. Previous systematic reviews highlighted peer support among mothers in maternity wards as a contributing factor to KMC acceptance [44]. While mother-initiated groups are less common in China, hospitals that invested in using social media for health promotion and establishing WeChat groups (a Chinese social media platform that enables group conversation) were perceived well among parents.

### Barriers and facilitators at parental level

In our study, we found mothers’ perception of the benefits of KMC to be an important factor in their willingness to provide KMC, this is consistent with previous studies [37]. During the interviews, many parents said that through skin to skin contact with their baby, they could feel the change of baby’s mood, and the babies became emotionally soothed. During skin-to-skin contact, parents felt they could “communicate” with their babies, which promoted the establishment of the parent-child relationship. We believe this is also part of the process of “humanizing care” for premature infants. KMC was also perceived to increase communication between parents and clinical staff, improving the often tense relationship between doctors and parents.

In our analysis, we identified a number of maternal individual factors that impeded KMC adoption. Some parents were affected by perfectionism and expressed stigma/fear/anxiety over their premature newborns especially when they are instructed to watch the monitors and notice changes in infants’ vital signs. Such negative emotions affect the establishment of bonding with infants, mothers cited difficulty in facing their premature babies. Clinical staff should look out for warning signs, encourage parents not to watch the monitors and offer support to parents by introducing them to peer support groups. Some parents were unable to provide KMC or could not continue with it for long due to pain, fatigue, complications and other physical factors, as also reported in previous studies [33, 34, 37, 45]. Therefore, providing comfortable chairs and a conducive environment for mothers could encourage parents to practice KMC for a longer time.

### Barriers and facilitators at financial level

Studies in low-income countries highlighted, commuting between home and KMC wards as a barrier to the provision of KMC. In cases where the newborn remained in the hospital after the mother was discharged, lack of money for transportation and distance to the hospital were reported as challenges to KMC implementation [46]. User fees for mother and baby to room-in for continuous KMC was considered a barrier in Indonesia, this was also an issue for some in our study. Our analysis indicated higher parental economic status as more conducive to the implementation of KMC, this is rarely mentioned in previous literature. Despite being a cost-effective intervention, performing KMC in China especially in the NICUs requires parents to travel to the hospital on a regular basis. Transportation costs or lodging costs to stay near the hospital might be burdensome for parents.

In addition, some nurses reported that inviting parents onto the NICU wards required additional hospital expenses as families would propose changing newborns diapers when nurses felt it was not needed. There was no charge for parents to be able to provide KMC in the hospitals participating in our study. Securing financial resources from hospital administration and/or parents medical insurance to facilitate KMC will be a necessary part of KMC scale-up in China.

### Recommendations

Based on the results of our analysis, we can draw the following recommendations relevant to others implementing or rolling out KMC at a national or sub-national level:

- An understanding of the specific barriers and challenges to implementing KMC in particular settings is fundamental to the success of implementation. Performing a barrier analysis prior to implementation is recommended.
- The support of hospital administration and leadership is crucial for the successful integration of KMC into routine practice. Support from hospital administrators and leaders means that space and resources can be made available for KMC provision and that staff received training in KMC, it also acts as a motivation for staff to promote KMC. Activities that increase the likelihood of hospital administrative support for KMC need to be included in KMC scale up plans, this could include targeting hospital administrators in awareness raising activities about the benefits and feasibility of KMC and the formation of a KMC network comprised of leaders from implementing hospitals.
- Staff motivation to offer and support the practice of KMC is vital for implementation. This support should be recognised as a key part of nursing care and considered by hospital administrators when assessing staffing levels and workload.
- Our findings and the findings of others highlight the impact of traditional cultural practices such as postpartum confinement (Zuo-yue-zi) on the uptake of KMC. Grandmothers and communities are perceived by mothers and health workers as key influencers of maternal practice [47]. This indicates a need for public promotion of KMC, potentially targeted at grandmothers alongside promotion in health facilities to be included in scale-up plans. Additionally, discharge education sessions for parents and grandparents should also include KMC and newborn danger signs monitoring and promote KMC continuum at home.
- Whilst maternal provision of KMC is preferable, primarily due to the establishment of breastfeeding, when a newborns mother is unavailable, this study has shown that paternal provision of KMC is feasible. It is recommended that fathers be engaged in the provision of KMC when mothers are unavailable.

### Strengths and limitations

This is the first multi-hospital qualitative study conducted in China on KMC adoption in hospital NICUs and postnatal wards. The five hospitals in the pilot study vary in geographic location, hospital specialty, local economic development, and prior to programme initiation had different levels of exposure to KMC knowledge and practice. While our study findings maybe specific to hospitals in these provinces, the summarized KMC barriers and facilitators could be similar to those of other provinces of China and could be useful to others initiating KMC programs.

Despite being the first study of its kind on KMC barriers and facilitators in China, several limitations should be considered. The perceived barriers and facilitators from parents’ perspectives might be biased as we only included parents who participated in KMC. We did not interview those who declined to provide KMC, those deemed not eligible by the hospital staff, or who did not have access due to the limited numbers of beds and rooms available for KMC in the hospitals. Reporting bias by medical staff due to social desirability was possible despite the research team repeatedly assuring the hospital staff that the interviews were not for service quality control. Many medical staff were also concerned about being judged for their knowledge of KMC (as trainings and tests happened often), but they usually appeared to relax after the first few questions; the private space and the guarantee of confidentiality also helped to ensure the authenticity of their answers.

## Conclusion

In this qualitative study, we analysed the perceptions of barriers and facilitators to KMC in China’s NICUs and postnatal wards, using data from observation and semi-structured interviews from five hospitals. We found barriers and facilitators existing in cultural, hospital, parental and financial levels. While several factors were common with other study settings including shortage of staff, increased workload and lack of space, we also discovered barriers and facilitators unique to the Chinese context, e.g. postpartum confinement, grandparents’ resistance and staff fear of nosocomial infection. We have recommended interventions targeting these barriers and facilitators including family and peer support for improved KMC adoption. The scale up of KMC in China could also contribute to the humanizing of neonatal care, with families becoming more involved in caring for the premature newborns. Additionally, for other countries and settings, we strongly recommend that specific barriers and challenges to KMC are identified and incorporated into scale up plans for KMC.

## Supplementary information

**Additional file 1.** Microsoft word document; Kangaroo mother care qualitative study field observation chart

**Additional file 2.** Microsoft word document; Kangaroo mother care qualitative study: semi-structured interview guide

**Additional file 3.** Microsoft word document; Kangaroo mother care qualitative study: detailed interviewee information

**Additional file 4.** Microsoft word document; Kangaroo mother care qualitative study: clinical observation information

## Data Availability

The datasets used and/or analysed during the current study are available from the corresponding author on reasonable request.

## Abbreviations

CFIR: Consolidated Framework for Implementation Research
KMC: Kangaroo mother care
NICU: Neonatal intensive care unit
PNW: Postnatal wards
WHO: World Health Organization
