# Supplementary material for: Barriers and facilitators of kangaroo mother care adoption in five Chinese hospitals: a qualitative study
Source: BMC Public Health. 2020 Aug 13;20:1234. doi: 10.1186/s12889-020-09337-6 (PMC7427278; doi:10.1186/s12889-020-09337-6)
Supplement: Supplementary file 1 — Additional file 1. Microsoft word document; Kangaroo mother care qualitative study field observation chart [file 12889_2020_9337_MOESM1_ESM.docx]

# **Additional file I. Kangaroo mother care qualitative study field observation chart**

| **KMC Observation Chart - NICU** | | | | |  |
| --- | --- | --- | --- | --- | --- |
| **Location:** | | | | | |
| **Observer:** | | | | | |
| Date: YYYY/MM/DD | | | | | |
|  | | | | | |
| **NICU Information** | | | | |  |
| Number of rooms __________  Number of beds____________  Number of patients _________  Number of doctors__________  Number of nurses___________  Doctor/bed ratio____________  Nurse/bed ratio_____________ | | | | |  |
| KMC implementation period and history | |  | | |  |
| Family visit (A: in-room; B: camera; C: corridor; D: others) | |  | | |  |
| Quarantine measure: required for family members when entering NICU:  A: hat; B: mask; C: gown; D: shoe cover; E: phone (allowed or not); F: others | |  | | |  |
| KMC chairs:  A: regular; B: reclining; C: others | |  | | |  |
| KMC gown: | |  | | |  |
| Note: | | | | |  |
| **Observation** | | | | |  |
| **Content** | **Observer:**  **Time:** | | **Observer:**  **Time:** | **Observer:**  **Time:** |  |
| Hand-washing pattern of doctors, nurses and family members before getting in touch with patients, before and after using phones |  | |  |  |  |
| Number of KMC conducted at present |  | |  |  |  |
| How medical staff assist with KMC |  | |  |  |  |
| Whether KMC and breastfeeding is recommended prior to discharge |  | |  |  |  |
| Feeding ingredient (multiple choice)  A: breastfeed; B: breast milk feed; C: donated breast milk; D: powder |  | |  |  |  |
| Feeding method (multiple choice)  A: breast feed; B: spoon and cup; C: bottle; D: tube |  | |  |  |  |
| Note: | | | | |  |

| **KMC Observation Chart – Postnatal Ward** | | | |
| --- | --- | --- | --- |
| **Location:** | | | |
| **Observer:** | | | |
| Date: YYYY/MM/DD | | | |
| **Postnatal Ward Information** | | | |
| Number of rooms __________  Number of beds____________  Number of preterm infants ___  Number of doctors__________  Number of nurses___________  Doctor/bed ratio____________  Nurse/bed ratio_____________ | | | |
| KMC implementation period and history |  | | |
| Accompany method (A: 24h; B: set time; C: others) |  | | |
| Note: | | | |
| **Observation** | | | |
| **Content** | **Observer:**  **Time:** | **Observer:**  **Time:** | **Observer:**  **Time:** |
| Hand-washing pattern of doctors, nurses and family members before getting in touch with patients, before and after using phones |  |  |  |
| Number of KMC conducted at the moment |  |  |  |
| Reasons for KMC pause over 15 minutes (observation only, infusion, rest, restroom, etc.) |  |  |  |
| How medical staff assist with KMC (position adjustment, indicator measurement) |  |  |  |
| Whether KMC and breastfeeding is recommended prior to discharge |  |  |  |
| Feeding ingredient (multiple choice)  A: breastfeed; B: breast milk feed; C: donated breast milk; D: powder |  |  |  |
| Feeding method (multiple choice)  A: breast feed; B: spoon and cup; C: bottle; D: tube |  |  |  |
| Family member that performed KMC (multiple choice) A: mother; B: father; C: others |  |  |  |
| KMC chairs:  A: regular; B: reclining; C: others |  |  |  |
| KMC gown: |  |  |  |
| Note: | | | |
|  |  |  |  |
|  |  |  |  |
|  |  |  |  |
