# Supplementary material for: Barriers and facilitators of kangaroo mother care adoption in five Chinese hospitals: a qualitative study
Source: BMC Public Health. 2020 Aug 13;20:1234. doi: 10.1186/s12889-020-09337-6 (PMC7427278; doi:10.1186/s12889-020-09337-6)
Supplement: Supplementary file 2 — Additional file 2. Microsoft word document; Kangaroo mother care qualitative study: semi-structured interview guide [file 12889_2020_9337_MOESM2_ESM.docx]

# **Additional file 2. Kangaroo mother care qualitative study: semi-structured interview guide**

**KMC interview guide for nurses and doctors**

**Personal information:** Age, sex, educational attainment, profession, title, years of working, KMC trainings received.

| Questions | CFIR Constructs |
| --- | --- |
| KMC Basic Information |  |
| - Do you know what’s KMC? - How does KMC influence premature infants’ growth? How does it influence parents? - What’s the procedure of KMC? | Knowledge and Beliefs About the Intervention |
| - Do you think KMC is complicated (duration, difficulty, procedure, charge)? | Complexity |
| - Who will you ask if you encounter any question? | Intervention Source |
| - Is KMC suitable for your daily work now? - How to integrate KMC into your daily work? - Does KMC promote your daily work? | Adaptability |
| - Is the workload of KMC one of your job performance criteria? - Has the lead doctor/nurse considered giving awards and recognition for KMC? | External Policy and Incentives |
| Examples of KMC |  |
| - Has anyone performed KMC today? - If yes, who made the decision to perform KMC? Why the infant was selected for KMC? Is there any other infant with similar condition that did not perform KMC? Why? - If no, why there’s no KMC performed today? When’s the last time KMC performed? | Intervention Source |
| KMC Training |  |
| - When and how did you first know of KMC? - How do the promotion materials influence you? - How was the training session? The pros and cons? Do you need more training? | Intervention Source  Knowledge and Beliefs About the Intervention |
| - At what stage are you aware of KMC intervention and utilization? | Individual Stage of Change |
| Resources Needed for KMC |  |
| - What basic infrastructure (design, equipment, personnel) will influence KMC implementation? | Structural Characteristics |
| - What do you think of the promotion materials (quality, clothing, mirror, cups, WeChat groups)? | Design Quality and Packaging |
| - What other resources are needed? | Patient Needs and Resources  Cost |
| - Do you think the current resource is adequate for KMC? | Patient Needs and Resources |
| - How to acquire those resources? What’s the challenge? - If inadequate, what other resource is needed from the hospital? Have you received any of those resource from hospital? What resource is easier to get? | Patient Needs and Resources |
| Acceptance among Hospital Colleagues and General Environment |  |
| - How do the hospital and ward leadership think of KMC? - What’s the goal for KMC implementation at the moment? How to achieve? - What’s the progress this month? Anyone in charge of data collection? - What’s the acceptance rate of KMC in the hospital and ward? Why? - Do you think your colleagues can assist parents to perform KMC? | Readiness for Implementation  Implementation Climate |
| - Does KMC provide comparative advantage for your hospital? | Peer Pressure |
| - What external policy could sustain KMC implementation? | External Policy and Incentives |
| - What’s the process of attitude change towards KMC? How did that happen? | Culture |
| Parents’ Feedback of KMC |  |
| - How many parents ask for KMC? How do they know of KMC? Wechat groups? - Why parents refuse KMC? Charge, location, emotion? - What’s their attitude towards KMC? - What’s parents’ understanding of KMC? - What’s parents’ feedback of KMC? Does that change doctor-patient relationship? | Patient Needs and Resources |
| - Do you have confidence for assisting parents to perform KMC? Why? | Self-Efficacy |
| Suggestions and Need for KMC |  |
| - During implementation, what changes are needed for KMC? - Do you think you can make those changes? Why and why not? | Patient Needs and Resources |
| - How to sustain the promotion of KMC in the wards? | Implementation Climate |
| - Do you think KMC should be scaled up? When should KMC be introduced to parents? Before or after delivery? | Implementation Climate |
| - Can you tell a story of collaboration with other departments/units during KMC implementation? | Networks and Communications |

Thank you for your participation!

**KMC parent interview guide**

**Personal information:** Age, sex, educational attainment, profession, relationship with the infant (mother, father, etc.), family condition (distance to the hospital, number of children, mode of delivery, etc.)

| Questions | CFIR Constructs |
| --- | --- |
| What is KMC? |  |
| - Do you know what’s KMC? | Knowledge and Beliefs About the Intervention |
| - How do you know of KMC? | Evidence Strength and Quality |
| - What influence your understanding of KMC? | Intervention Source |
| - How does KMC influence premature infants’ growth? How does it influence you? Why and why not? | Knowledge and Beliefs About the Intervention  Peer Pressure |
| - Some says KMC is useful and some doesn’t, what’s your attitude towards KMC? (Relevant? enthusiastic? Not optimistic?) Why? | Knowledge and Beliefs About the Intervention |
| Examples of KMC |  |
| - Has anyone in the ward performed KMC today? If yes, who made the decision to perform KMC? - Why KMC was decided to be used and why not? | Intervention Source |
| - How do you perform KMC? Please consider these about KMC: should it be charged? skin contact (skin-to-skin or over clothes), duration, complicated or not, procedure, intervention, etc. | Complexity |
| - What daily changes are needed for KMC? Do you think you can make those changes, why and why not? - How to integrate KMC into your daily life? What’s the challenge? | Adaptability |
| KMC Guidance and Promotion |  |
| - How do you evaluate the guidance received? - How did the guidance assist KMC implementation? Can you explain? - What’s the pros and cons of KMC guidance? - Do you need more guidance? - Who will you ask if you have questions for KMC? | Intervention Source |
| - How do you think of KMC promotion materials? When is the best time to introduce KMC to you? (If there’s a WeChat group? How the group is managed? How’s the quality of the promotion material?) - What are the other ways that KMC could be promoted? E.g. WeChat groups, websites? | Design Quality and Packaging |
| - Can you describe how do you cooperate with medical staff during KMC? Do you think KMC influence your relationship with doctors and nurses? - Can you tell me an actual case? | Networks and Communications |
| - How confident are you in terms of performing KMC? - What makes you more or less confident? | Self-Efficacy |
| Resources Provided for KMC |  |
| - Does the hospital provide adequate resource (e.g. bed, clothes, bandage, glass, cups) for you to perform KMC? - [If yes] how do you acquire such resources? How do you use them? What other resources are needed but hard to acquire? - [If not] what are the resources/time needed? | Patient Needs and Resources |
| - Do you have enough time to perform KMC? | Patient Needs and Resources |
| - What other basic infrastructure (design, equipment, personnel) will influence KMC implementation? | Structural Characteristics |
| Family Support and Collaboration |  |
| - How do other family members think of KMC? Do they support KMC? Why? | Evidence Strength and Quality |
| - Can you tell me how do you collaborate with other family members in KMC? - Can you tell an actual case? | Networks and Communications  Implementation Climate |
| - What family condition will influence KMC implementation? | Structural Characteristics |
| Feedback from other Parents |  |
| - How many other parents ask for KMC? How do they know of KMC? | Patient Needs and Resources |
| - How was the WeChat group established? | Patient Needs and Resources |
| - Why other parents refuse to perform KMC? What’s their perception of KMC? | Patient Needs and Resources |
| - Have you heard of other parents performing KMC? - Can you tell an actual case? | Patient Needs and Resources |
| What stage are you in terms of understanding of KMC? (Choose one) |  |
| - Understanding: Heard of KMC, want to know more of KMC? - Convinced and considering: Accept KMC and would like to discuss using it - Decided to use: Determined to perform KMC - Using periodically: Have knowledge of KMC and using it periodically - Using regularly: Understand KMC’s benefit and use it regularly, and plan to promote it to other people | Self-efficacy  Individual Stage of Change |

Thank you for your participation!
