# Supplementary material for: Barriers and facilitators of kangaroo mother care adoption in five Chinese hospitals: a qualitative study
Source: BMC Public Health. 2020 Aug 13;20:1234. doi: 10.1186/s12889-020-09337-6 (PMC7427278; doi:10.1186/s12889-020-09337-6)
Supplement: Supplementary file 3 — Additional file 3. Microsoft word document; Kangaroo mother care qualitative study: detailed interviewee information [file 12889_2020_9337_MOESM3_ESM.docx]

# **Additional file 3. Kangaroo mother care qualitative study: detailed interviewee information**

**eTable 1.** **Characteristics of the medical staff interviewed**

| **ID** | **Hospital** | **Type** | **Dept** | **Education** | **Rank** | **Tenure** | **Training** |
| --- | --- | --- | --- | --- | --- | --- | --- |
| 1 | A | Nurse | Obstetrical | Bachelor | Senior | 14 | Y |
| 2 | A | Nurse | Pediatric | Bachelor | Junior | 4 | Y |
| 3 | A | Doctor | Obstetrical | Master | Senior | 16 | N |
| 4 | A | Doctor | Pediatric | Master | Senior | 28 | N |
| 7 | A | Nurse | Obstetrical | Bachelor | Senior | 14 | Y |
| 8 | A | Nurse | Obstetrical | College | Junior | 3 | Y |
| 9 | A | Nurse | Pediatric | Bachelor | Junior | 6 | Y |
| 10 | A | Nurse | Pediatric | Bachelor | Junior | 8 | Y |
| 11 | B | Nurse | Pediatric | Master | Junior | 8 | Y |
| 12 | B | Nurse | Pediatric | Bachelor | Senior |  | Y |
| 13 | B | Doctor | Pediatric | Doctorate | Junior | 5 | Y |
| 14 | B | Doctor | Pediatric | Doctorate | Junior | 3 | Y |
| 17 | C | Nurse | Pediatric | Bachelor | Junior | 9 | Y |
| 18 | C | Nurse | Pediatric | Bachelor | Senior | 10 | Y |
| 19 | C | Doctor | Pediatric | Master | Junior | 1 | Y |
| 20 | C | Doctor | Pediatric |  | Senior | 17 | N |
| 23 | D | Nurse | Pediatric | Bachelor | Senior | 9 | Y |
| 24 | D | Nurse | Pediatric | Bachelor | Senior | 12 | Y |
| 25 | D | Doctor | Pediatric | Master | Junior | 6 | Y |
| 26 | D | Nurse | Pediatric | Bachelor | Senior | 28 | Y |
| 27 | D | Nurse | Obstetrical | Bachelor | Senior | 14 | Y |
| 28 | D | Nurse | Obstetrical |  | Junior | 4 | Y |
| 29 | D | Doctor | Obstetrical | Bachelor | Junior | 6 | Y |
| 30 | D | Nurse | Obstetrical | Bachelor | Senior | 18 | Y |
| 33 | E | Nurse | Pediatric | Bachelor | Junior | 9 | Y |
| 34 | E | Nurse | Pediatric |  | Senior | 23 | Y |
| 35 | E | Doctor | Pediatric | Master | Senior | 16 | N |
| 36 | E | Doctor | Pediatric | Bachelor | Junior | 7 | Y |

**eTable 2. Characteristics of the parents interviewed**

| **ID** | **Hospital** | **Education** | **Job** | **No. of baby** | **Type of family** |
| --- | --- | --- | --- | --- | --- |
| 5 | A |  | Unemployed | 1 | Extended family |
| 6 | A | College | Business | 2 | Extended family |
| 15 | B | College | Unemployed | 2 | Nuclear family |
| 16 | B | Bachelor | Teacher | 1 | Nuclear family |
| 21 | C | Junior high school | Beautician | 2 | Extended family |
| 22 | C | Junior high school | Driver | 1 | Nuclear family |
| 31 | D | College | Sales | 2 | Extended family |
| 32 | D | High school | Unemployed | 4 | Nuclear family |
| 37 | E | College | Unemployed | 2 | Nuclear family |
| 38 | E | Bachelor | Office clerk | 1 | Extended family |
